# Association between Variation of Troponin and Prognosis of Acute Myocardial Infarction before and after Primary Percutaneous Coronary Intervention

**DOI:** 10.1155/2020/4793178

**Published:** 2020-07-25

**Authors:** Xiaoxiao Zhao, Ying Wang, Chen Liu, Peng Zhou, Zhaoxue Sheng, Jiannan Li, Jinying Zhou, Runzhen Chen, Yi Chen, Hanjun Zhao, Hongbing Yan

**Affiliations:** ^1^Department of Cardiology, Fuwai Hospital, National Center for Cardiovascular Diseases, Peking Union Medical College, Chinese Academy of Medical Sciences, Beijing, China; ^2^Fuwai Hospital Chinese Academy of Medical Sciences, Shenzhen, China

## Abstract

**Background:**

Circulating levels of cardiac troponin I (cTnI) after ST-segment elevation myocardial infarction (STEMI) were considered as prognostic factors for predicting the incidence of major adverse cardiovascular events (MACE). △cTnI is the difference between peak cTnI after primary percutaneous coronary intervention (PPCI) and cTnI on initial admission.

**Purpose:**

This study aimed to assess the relationship between △cTnI, the ratio of △cTnI to cTnI on initial admission, and the incidence of MACE during the follow-up period.

**Methods:**

A total of 2596 patients with cTnI measured upon admission and one-time measurement of cTnI during hospitalization were enrolled.

**Results:**

In the adjusted models of the survival receiver operating characteristic (ROC) curve, △cTnI and the ratio of △cTnI to cTnI on initial admission have stronger discrimination power of MACE (area under curve (AUC) 0.730 and 0.717) compared with peak cTnI after PPCI and cTnI at admission (AUC 0.590, 0.546). Multivariate Cox regression analysis identified △cTnI (hazard ratio (HR) 1.018, 95% confidence interval (CI) 1.001 to 1.035) as a relevant factor for MACE during follow-up. △cTnI was divided into quartiles, and maximum △ cTnI between 4.845 and 19.073 ng/ml comprised more patients with anterior wall myocardial infarction (*p* < 0.001), higher GRACE score (*p* = 0.038), CK-MB (*p* = 0.023), and Myoglobin (*p* < 0.001). On the K–M survival curves, the incidence of MACE, mortality, and angina pectoris were significantly higher in the group with maximum △cTnI (*p* = 0.035, 0.049, 0.026).

**Conclusion:**

The △cTnI level and the ratio of △cTnI have stronger discrimination power of predicting the incidence of MACE. The group with maximum △cTnI has higher incidence of MACE, mortality, and angina pectoris during the follow-up period.

## 1. Introduction

Cardiac troponin I (cTnI) is a highly specific and sensitive biomarker of cardiac injury and a regulatory protein with cytosolic and structural compartments within the cardiac myocytes [1]. cTnI is a heart-specific protein released in the circulation upon myocardial injury and plays a significant role in the regulation of muscle contraction and cardiac troponins [2]. Conventional assay measurements of cTnI levels are routinely used to rule out acute myocardial infarction (AMI) and to assess the 30-day and 90-day prognoses of patients presenting with acute coronary syndrome (ACS) [3, 4]. Various studies [5–7] have reported that the circulating levels of cTnI after ST-segment elevation myocardial infarction (STEMI) are related to clinical outcomes and considered a prognostic predictor of major adverse cardiovascular events (MACE). However, the relationship of the cTnI level difference between the pre- and post-primary percutaneous coronary intervention (PPCI) is not well defined. To address this knowledge gap, this study aimed to explore the prognostic value of the cTnI level difference between PPCI peak cTnI and first admission cTnI to MACE during follow-up in a contemporary, homogeneous, and well-defined cohort of patients with STEMI undergoing PPCI. We investigated the correlation of first cTnI, peak cTnI after PPCI, △cTnI, and the ratio of △cTnI to cTnI on initial admission, considering coronary angiography and echocardiography data and incidence of MACE. Moreover, we compared the discriminatory value of all four parameters in discriminating MACE.

## 2. Methods

### 2.1. Subjects

From a total of 4064 patients who presented at Fuwai Hospital in Beijing, China, between January 2010 and July 2018, 3586 consecutive STEMI patients (2713 men; age: 24–97 years) were enrolled (478 patients who were lost to follow-up were excluded from the study). All patients were referred to the coronary catheterization center with the diagnosis of acute STEMI fulfilling the criteria for PPCI according to the guidelines [8, 9]. The study was approved by the Ethics Committee of Fuwai Hospital, and all patients gave informed consent for coronary angiography and PPCI.

**Figure 1:**
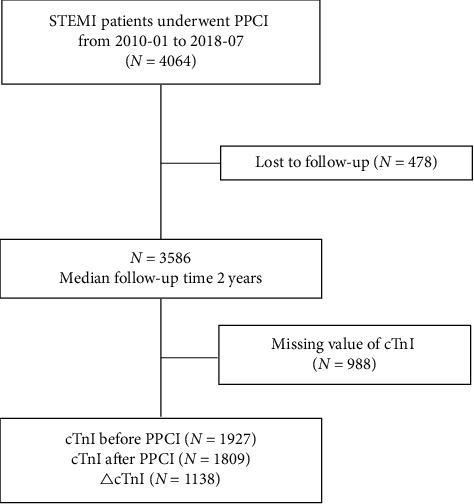
Flow chart of study enrollment. cTnI, cardiac troponin I; PPCI, primary percutaneous coronary intervention; STEMI, ST-segment elevation myocardial infarction.

Patient records including demographics, medical history, physical examination, blood test results, electrocardiography (ECG), echocardiography data, and discharge medication regimen were reviewed. Blood testing was performed at the clinical laboratory in Fuwai Hospital. Blood samples for cTnI measurement were acquired on admission and after PPCI. Patients were required to have at least one measurement of the cTnI level during hospitalization. However, of the 3586 patients, those who did not have a valid cTnI result and those with missing postdischarge follow-up data were also excluded. Finally, 2598 patients were included in the analysis. The study flow chart is shown in Figure 1.

### 2.2. Definitions

In this study, △cTnI was defined as the value calculated as the post-PPCI peak cTnI value minus the first admission cTnI value. STEMI was defined as continuous chest pain lasting >30 min, an elevated troponin I level, and an ECG finding of ST-segment elevation >0.1 mV in at least two contiguous leads or a new left bundle-branch block on an 18-lead ECG [10]. Hypertension was defined as a blood pressure ≥140/90 mmHg in three occasions at rest or previous diagnosis of hypertension and current use of antihypertensive drugs. Diabetes mellitus (DM) was defined according to the 75 g oral glucose tolerance test (OGTT); that is, patients were diagnosed with DM if they met one of the following criteria: (i) a fasting plasma glucose level of ≥7.0 mmol/L, (ii) a 2 h value of ≥11.1 mmol/L in 75 g OGTT, and (iii) a casual plasma glucose level of ≥11.1 mmol/L. Dyslipidemia was defined by any of the following parameters: the total cholesterol (TC) 5.0 mmol/L, low-density lipoprotein cholesterol (LDL-C) ≥3.0 mmol/L, triglycerides (TG) ≥1.7 mmol/L, high-density lipoprotein cholesterol (HLH-C) ≥ 1.2 mmol/L (women) or ≥ 1.0 mmol/L (men), or statin treatments. Height and weight were measured by trained medical staff; the body mass index was calculated by weight (kg)/height squared (m^2^). The no-reflow phenomenon was defined as thrombolysis in a myocardial infarction (TIMI) flow grade <3 after PPCI.

### 2.3. Biomarker Measurements

Blood samples were drawn from an antecubital vein in the morning after overnight fasting and collected into vacuum tubes containing EDTA for the measurement of plasma lipid and lipoprotein levels. TC, (HDL-C), LDL-C, TG, and homocysteine levels were analyzed by colorimetric enzymatic assays using an auto analyzer at the chemistry laboratory of the Fuwai Hospital Peking of Union Medical College. The level of C-reactive protein (CRP) was measured by biodirectional lateral flow immunoassay according to the procedure at the same chemistry laboratory.

Myocardial biomarkers were measured as follows: cTnI levels were measured on blood samples collected upon admission and after PPCI and serum was separated internally. The Abbott ARCHITECTi2000SR (Hong Kong, China) immunoassay system (batch number: 73099UI00) and Beckman UniCelDXI800 (California, USA) access immunoassay system (batch number: 624362) were used to analyze the cTnI level. The detection limit of the assay is 0.02 ng/ml, and the decision limit for the diagnosis of MI is 0.07 ng/ml.

### 2.4. Primary Stenting and Antiplatelet Therapy

PPCI (stenting/balloon dilatation/thrombus aspiration) was performed using standard criteria. Heparin or bivalirudin was used as periprocedural anticoagulant therapy. Glycoprotein IIb/IIIa inhibitors were used at the discretion of the operator. Commercially available stents were used. The dual antiplatelet therapy following PPCI consisted of oral aspirin (80–325 mg/day continued indefinitely) and a P2Y12 inhibitor for at least 12 months. Other medications were prescribed at the discretion of the trained attending physicians.

### 2.5. ECG and Echocardiography Collection

All ECG tracings from the emergency response teams and emergency department were collected and analyzed. Additional 18-lead ECGs were obtained upon arrival to the emergency department and twice daily thereafter until discharge. Each participant was scanned by trained ultrasonographers using the color Doppler ultrasonic diagnostic apparatus, cardiac ultrasonic measurement software, and a linear array transducer at a frequency of 12 MHz.

### 2.6. MACE and Follow-Up

For adverse events that occurred at follow-up, the following were evaluated: overall mortality, recurrence of MI (beyond 96 h of hospitalization, defined by the recurrence of chest pain accompanied by either re-ST-segment elevation as described above or ST-segment depression attributed to myocardial ischemia and re-elevation of cTnI > 25%), and stroke.

### 2.7. Statistical Analysis

The normal distribution of outcome variables was confirmed by Kolmogorov–Smirnov tests. Baseline parameters and major adverse events during follow-up were presented as median (interquartile range) for continuous variables and as frequency and percentage for categorical variables. The relationship between baseline clinical, ECG, angiography characteristics, follow-up outcomes, and peak levels was assessed by the Spearman correlation. Survival analysis was performed with the Kaplan–Meier method. To assess the discrimination utility of cTnI, we plotted the time-dependent receiver operating characteristic (ROC) curves conducted by R language in order to obviate the limitation of potentially biased due to censoring. The predictive values of the ratio of delta cTnI to cTnI measured prior PPCI, delta cTnI, peak cTnI level post-PPCI, and the first admission cTnI level were obtained in the range of 0-1 using the logistic regression model by controlling the following: history of hypertension, DM, hyperlipidemia, coronary artery bypass grafting, PCI, smoking, chest pain onset to hospital stay, Apo A, Apo B, CRP, TC, TG, HDL-C, LDL-C, and medication history (aspirin, clopidogrel, warfarin, angiotensin-converting enzyme inhibitor, angiotensin receptor blockers, beta receptor blocker, statin, enzyme, and nitrates). The ROC curves were obtained by incorporating three predictive values. We tabulated the baseline characteristics of the cohort and, then, examined the bivariate association between these variables and △cTnI quartiles. Differences across △cTnI quartiles were evaluated by the analysis of variance (normally distributed variables) or Kruskal–Wallis test (skewed variables) for continuous variables and with the *χ*2 test for categorical variables. Univariable and multivariable Cox proportional hazards regression modeling was performed to characterize predictors of MACE. Categorical variables included the △cTnI group, target lesion types of culprit vessels of MI, status of target organ thrombosis, status of the target organ occlusion, and whether the target lesion involves branches. Continuous variables included the △cTnI level difference before and after PPCI and the GRACE score. Significant variables analyzed were reported with their respective hazard ratios and 95% confidence limits. All *p* values are two-tailed, and statistical significance was determined at *p* < 0.05. Time-dependent ROC curves were performed with R language version i386 3.6.2. The other analyses were performed with SPSS version 20.0 statistical software (SPSS, Inc., Chicago, IL).

## 3. Results

### 3.1. Patient Demographics

**Figure 2:**
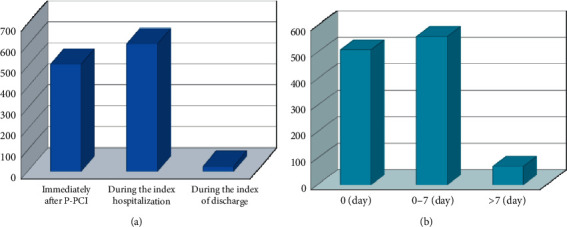
The time of the second sample taken after primary PCI. There were 509 (44.728%) cases measured immediately after PPCI and 606 (53.251%) measured during the index hospitalization. Five hundred and nine (44.728%) cases were measured at the same day of primary PCI and the cases measured during 0–7 days were 560 (49.209%).

The median (interquartile range) time between symptom onsets to admission was 7 h (8 h). Patients' baseline clinical characteristics are summarized in Table 1. During the median 2-year follow-up, 293 patients (8.2%) died (170) and other 68 patients had stroke. The mean age of the cohort was 59 years, and 75.5% were men (Table 1). Moreover, 53.1% of patients were current smokers. The prevalence rates of hypertension, hyperlipidemia, and DM were 59.6%, 77.6%, and 32.5%, respectively. We compared the baseline characteristics of patients excluded from the analysis due to the lack of repeated troponin assessments versus patients included in the analysis in Table 2. There was no significant difference between two groups in the variables of the age, BMI, heart rate, systolic blood pressure, the history of PCI, history of CABG, the prevalence of hypertension, diabetes, the use of aspirin, warfarin, angiotensin receptor blocker, ezetimibe, and the incidence of stroke after follow-up.  Figure 2 showed the time of the second sample taken after primary PCI. There were 509 (44.728%) cases measured immediately after PPCI and 606 (53.251%) measured during the index hospitalization. On the other hand, we found that 509 (44.728%) cases measured immediately after PPCI and the cases measured during 0–7 days were 560 (49.209%). Furthermore, we compared the peak troponin level with the delta troponin level and significant difference between two groups (*p* < 0.001).

### 3.2. Effects on Cardiac Troponin Elevation Kinetics

Correlation coefficients between cTnI and angiography characteristics, outcome at 2-year follow-up, and echocardiography measurement at discharge are shown in Table 3. Patients were categorized into three groups: cTnI levels upon admission (first cTnI), peak cTnI levels after PPCI (peak cTnI post-PPCI), and difference in cTnI between pre- and post-PPCI (△cTnI). Table 3 presents the independent significantly positive correlation between △cTnI and type of the target lesion (coefficient = 0.091, *p* = 0.005), status of target organ thrombosis (coefficient = 0.154, *p* ≤ 0.001), status of target organ with complete occlusion (coefficient = 0.203, *p* ≤ 0.001), left atrial diameter (LAD) on discharge (coefficient = 0.145, *p* ≤ 0.001), LVEDV on discharge (coefficient = 0.139, *p* = 0.0050), and mortality on the median 2-year follow-up (coefficient = 0.438, *p* ≤ 0.001). Moreover, an independent significantly negative correlation was found between △cTnI and the time from symptom onset to admission (coefficient = −0.161, *p* ≤ 0.001), TIMI flow grade pre-PPCI (coefficient = −0.214, *p* ≤ 0.001, minimum vessel diameter (coefficient = −0.214, *p* ≤ 0.001), and LVEF on discharge (coefficient = −0.019, *p* ≤ 0.001).

### 3.3. Discrimination of the Value of the Ratio of Delta cTnI to cTnI Measured Prior PPCI, △cTnI, Peak cTnI after PPCI, and First cTnI

**Figure 3:**
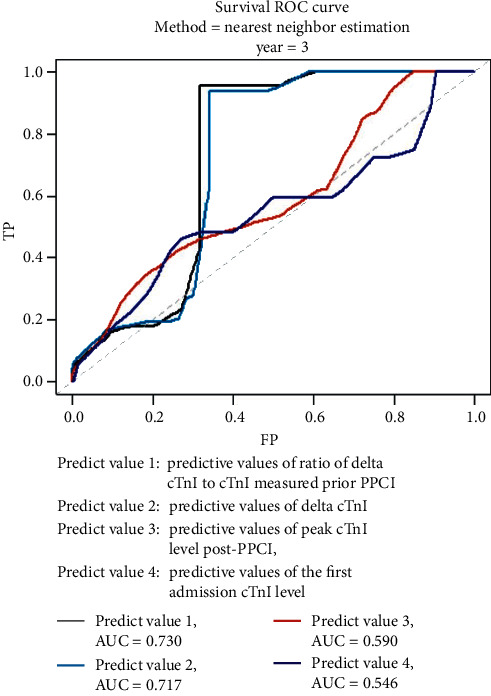
Survival ROC curve. Predictive value 1: predictive values of ratio of delta cTnI to cTnI measured prior PPCI; predictive value 2: predictive values of delta cTnI; predictive value 3: predictive values of the peak cTnI level after PPCI; and predictive value 4: predictive values of the first admission cTnI level. Survival receiver operating characteristic curves for a model including the ratio of delta cTnI to cTnI measured prior PPCI (black line), △cTnI level (wathet blue line), peak level after PPCI (red line), and cTnI concentrations at admission (dark blue line). The areas under the ROC curve are shown for the graph. AUC, area under the curve; ROC, survival receiver operating characteristic; TP, true positive; and FP, false positive.

Figure 3 shows the survival (time-dependent) ROC curves for the discrimination value of MACE of the ratio of delta cTnI to cTnI measured prior PPCI, △cTnI, peak cTnI after PPCI, and first cTnI. The areas under the ROC curve (AUC) are 0.730, 0.717, 0.590, and 0.546, respectively.

### 3.4. Stratified Analysis by the △cTnI Level Difference between Pre- and Post-PPCI

The characteristics of the group with the maximum difference (fourth group) in cTnI levels (ng/mL) that ranged from 19.161 to 101.66 (median value 52.00) at baseline, as shown in Table 4, are as follows. The group with the maximum △cTnI level between 4.845 ng/ml and 19.073 ng/ml was composed of patients with anterior wall myocardial infarction (*p* < 0.001), higher Global Registry of Acute Coronary Events (GRACE) score (*p* = 0.038), creatine kinase MB (*p* = 0.023), and myohemoglobin (*p* < 0.001), while age, sex, the no-reflow phenomenon, triple-vessel disease, the number of stents, and risk factors including hypertension, hyperlipidemia, and DM failed to present statistical difference between groups (Table 4). Multivariate Cox regression analysis identified the △cTnI level (HR: 1.018, 95%, CI: 1.001–1.035, *p* = 0.042), Q2 group (HR: 4.080, 95% CI: 1.342–2.403, *p* = 0.013), and uric acid (HR: 0.0 = 987, 95% CI: 0.977–0.988, *p* = 0.018) as relevant factors for MACE during follow-up (Table 5).

### 3.5. Survival Analysis of the △cTnI Level Difference before and after PPCI

**Figure 4:**
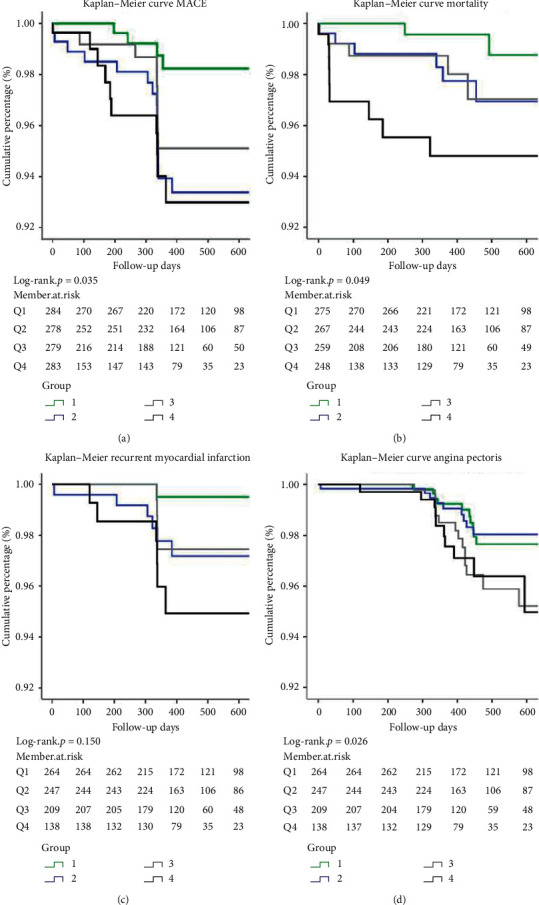
Cumulative incidence of myocardial infarction or cardiac death in patients with troponin concentrations. Patients were stratified into four groups based on the △cTnI level. Q1: first quartile (green line); Q2: second quartile (blue line); Q3: third quartile (grey line); and Q4: fourth quartile (black line). (a) Kaplan–Meier curves depicting the cumulative probability for MACE (log rank *p* = 0.035). (b) Kaplan–Meier curves depicting the cumulative probability for mortality (log rank *p* = 0.049). (c) Kaplan–Meier curves depicting the cumulative probability for recurrent myocardial infarction (log rank *p* = 0.150). (d) Kaplan–Meier curves depicting the cumulative probability for angina pectoris (log rank *p* = 0.026). MACE: major adverse cardiovascular events.

The median follow-up time was 2 (range <1–8.35) years. The Kaplan–Meier curves depicted a cumulative probability of MACE, mortality, recurrent myocardial infarction, and angina pectoris for patients stratified into quartiles of the △cTnI level at enrollment (log rank *p* = 0.035, 0.049, 0.015, 0.026) (Figure 4).

## 4. Discussion

This study investigated defined variables in a large-scale, well-described, and contemporary-treated STEMI population from China. The novelty of the study was the assessment of the discrimination value of the △cTnI and the ratio of △cTnI to cTnI on initial admission to the incidence of MACE which remains largely unknown. The researchers followed strict inclusion and exclusion criteria, which facilitated a reasonably streamlined and comparable hospital flow for all subjects. On the other hand, the use of time-dependent ROC curves may obviate potentially biased due to censoring.

### 4.1. Effects on High △cTnI Kinetics

Several prior investigations supported that the early release of cTnI following AMI is caused by the washout mechanism from the infarcted zone into the serum, a process which is facilitated by early restoration of blood flow to the infarcted tissue [11–13]. In this study, the analyzed patients were selected from a large retrospective cohort of 3586 patients with STEMI undergoing contemporary primary percutaneous revascularization and supported the independent significantly positive correlation between △cTnI and the type of the target lesion, status of target organ thrombosis, status of target organ with complete occlusion, LAD on discharge, LVEDV on discharge, and mortality on the median 2-year follow-up. Several previous studies [14–18] have shown that cardiac troponin could predict the LV function after STEMI patients were reperfused pharmacologically. Moreover, we found that peak troponin concentration after PPCI was associated with the TIMI flow grade at pre-PPCI (coefficient = −0.206, *p* ≤ 0.001), minimum vessel diameter prior PPCI (coefficient = −0.065, *p* = 0.010), LAD on discharge (coefficient = 0.135, *p* ≤ 0.001), LVEDV on discharge (coefficient = 0.116, *p* ≤ 0.001), and LVEF on discharge (coefficient = −0.156, *p* ≤ 0.001).

### 4.2. △cTnI and the Ratio of △cTn Have a Discriminative Value of Follow-Up Outcomes

Many studies reported [19–22] that an increased first cTnI level and peak cTnI were associated with an adverse outcome of primary angioplasty in AMI. Matetzky et al. [23] found that, in AMI patients with ST-segment elevation, an elevated cTnI on admission was associated with an increased risk of primary angioplasty failure and a more complicated clinical course. Testa [24] reported that a small increase in troponin concentration after a successful elective PCI was not infrequent and did not affect the outcome. Our study focused on the relationship between △cTnI between post-PPCI peak cTnI and first cTnI and comprehensive 2-year outcome assessments in PPCI-treated STEMI patients, which are an important addition to the current knowledge, as similar studies on the era remain limited. We found that the group with the maximum △cTnI level between 4.845 and 19.073 (median 52.00) has a statistically higher number of patients with target lesion thrombosis (*p* = 0.002), complete occlusion of the target lesion (*p* ≤ 0.001), TIMI blood flow grade 0 at pre-PPCI (*p* ≤ 0.001), and statistically significant lower use of cardiovascular-related drugs at post-PPCI than other groups. Based on the Kaplan–Meier analysis of the probability of death, the mortality rate of the group with the maximum △cTnI (median 52.00) was significantly higher than that of other groups.

### 4.3. Characteristics of the Group with the Maximum △cTnI Level

Sezer et al. [25] reported that, in patients with anterior wall AMI treated with PPCI, absolute and relative neutrophilia and mean platelet volume were independently associated with impaired microvascular perfusion. Kobayashi et al. [26] observed that a wraparound LAD predicted adverse clinical outcomes (hazard ratio: 2.18, *p* = 0.02) and severe heart failure (odds ratio 3.31, *p* = 0.049) at 3 years in patients with anterior STEMI who underwent PPCI. These observations, together with the present results, tend to support the characteristics of the fourth group of the △cTnI level with the maximum cases of anterior wall MI and higher risk for MACE. The pathophysiology of the no-reflow phenomenon is complex, and a series of consistent data has [27, 28] clearly shown that the no-reflow phenomenon has a strong negative effect on the outcome. In a population-based global registry, the NCDR study reported [29] that the no-reflow phenomenon is associated with an increased risk of adverse postprocedure hospital course including higher in-hospitality mortality (6.8% vs. 2.9%; *p* = 0.01), cerebrovascular accident (1.5% vs. 0%; *p* < 0.001), postprocedure bleeding (2.3% vs. 0.5%; *p* = 0.009), and cardiogenic shock (3.8% vs. 1.2%; *p* = 0.011). The no-reflow phenomenon is a process in which prolonged ischemia caused changes in endothelial cells, and optimal treatment of hyperglycemia is a significant target in preventing [30, 31]. On the contrary, we did not find a significant statistical difference in the no-reflow phenomenon between the four groups according to △cTnI at pre- and post-PPCI (*p* = 0.337). This was possibly caused by the number of participants and the rare occurrence of the no-reflow phenomenon after PPCI in our study.

The findings from studies [32, 33] emphasized the counterbalancing effects of ischemic and hemorrhagic complications after stent implantation and revealed the significant effect of the stent on patient outcomes. The analysis of the characteristics of the fourth group with poor prognosis of MACE failed to determine that the number of stents (*p* = 0.272) and triple-vessel disease (*p* = 0.984) have a significant statistical difference with other groups. Because of the inadequate calibration, the actual prevalence of the four groups according to the △cTnI level fluctuation may need to be viewed with caution in Chinese population with STEMI following PPCI. Several studies have reported the predictive accuracy of the GRACE risk score in different patient populations. A study of contemporary populations [34] with a validated GRACE risk score suggested that the GRACE risk score could predict in-hospital and 6-month mortality and has high collinearity between LVEF in a cohort of patients with ACS. The study [35] concluded that the GRACE risk score demonstrated a significant discriminatory ability for adverse outcomes. In conclusion, these results indirectly support that the statistically significant higher GRACE risk score in the fourth △cTnI group has contributed to the higher incidence of MACE (*p* ≤ 0.001). Furthermore, mathematical models and artificial intelligence have recently come in help in the setting, and integration into the clinical workflow might significantly improve patient outcomes [36]. The novel dynamical model synthetically describes the basic mechanisms underlying cTnI release into the plasma after the onset of AMI which provide the clinicians with a quantitative tool to analyze the series.

### 4.4. Limitations

Nevertheless, this study has several potential limitations. Firstly, it is a single-center, retrospective study design with strict inclusion criteria, and many patients who did not have a valid cTnI result were excluded, which resulted in selection bias. Secondly, patients have been enrolled during a long span of time, which could bring about a confounding effect due to the improvements of interventional techniques and progress in medication. Multicenter studies with a larger sample of patients admitted in a short period would be preferred to validate the results of this study. While the discrepancies in discharge medication were explored, a potentially unbalanced distribution of missing values may have influenced the results. Finally, we did not analyze some acute phase biomarkers such as ST-segment recovery and reperfusion ventricular arrhythmia “bursts” which are also related to the outcome in our models. Thus, the relative predictive information provided by △cTnI simultaneously considered with such additional biomarkers remains an important area for future research.

## 5. Conclusions

The main findings of this study are as follows: (1) △cTnI and the ratio of △cTnI to cTnI on initial admission were significant prognostic indicators in patients with MACE compared with first cTnI and peak cTnI after PPCI. (2) The maximum △cTnI level (median 52.00) was associated with a higher incidence of MACE, mortality, and angina pectoris at media 2-year follow-up than the other groups. (3) A higher number of patients with the maximum △cTnI level more likely had anterior wall MI (*p* < 0.001), a higher GRACE score (*p* = 0.038), myohemoglobin (*p* < 0.001), and TIMI flow grade 0 at pre-PPCI (*p* < 0.001).

Our findings supported that repeated measurements of cTnI and △cTnI could provide significant incremental information for risk stratification of STEMI patients who underwent PPCI. It represents a valuable, inexpensive, and readily available tool for assessment of risk, especially of long-term MACE; in addition, our results suggest that routine use of △cTnI values provide complementary information to well-established clinical risk factors. Furthermore, one can envision a △cTnI biomarker-guided approach to implement aggressive medical treatment strategies before discharge in patients at increased risk. Finally, it could provide help in the future selection of individuals considered for inclusion in clinical trials aimed at improving outcomes in such a population.

## Figures and Tables

**Table 1:** Baseline characteristics of the study cohort.

	All enrolled	cTnI pre-PPCI cohort	Cohort of the maximum troponin in measurement data	△cTnI cohort
N	3586	1927	1809	1138
Age (years)	59 (16)	58 (18)	57 (19)	59(12.31)
Male (%(*n*))	75.7 (2713)	73.3 (1412)	72.2 (1306)	68.5 (779)
Time from the onset of symptoms to admission (h)	7.00 (8.00)	7.00 (9.00)	7.00 (8.00)	7.00 (9.00)
Time from the onset of symptoms to balloon (h)	15.97 (18.43)	15.58 (23.90)	14.86 (25.58)	14.38 (20.46)
BMI (kg/m^2^)	25.76 (4.48)	25.26 (5.10)	25.16 (5.46)	25.99 (3.76)
Heart rate (beats per minute)	76 (19)	75 (18)	75 (18)	77 (36)
Systolic blood pressure (mmHg)	122 (25)	123 (24)	123 (24)	124 (40)
Diastolic blood pressure (mmHg)	74 (16)	75 (17)	75(16.5)	77 (25)
History of PCI (%(*n*))	11.6 (417)	11.4 (220)	10.4 (189)	9.8 (111)
History of CABG (%(*n*))	0.8 (30)	0.9 (17)	0.8 (15)	0.9 (10)
*Risk factors*				
Hypertension (%(*n*))	59.6 (2136)	59.1 (1138)	60.1 (1088)	59.3 (675)
Hyperlipidemia (%(*n*))	77.6 (2782)	71.6 (1380)	71 (1285)	64.9 (739)
Smoking (%(*n*))	53.1 (1903)	46.7 (900)	45.8 (828)	38.5 (438)
Diabetes (%(*n*))	32.5 (1165)	33.1 (638)	31.9 (577)	32.2 (366)
*Laboratory examinations*				
cTnI (ng/ml)	—	0.37 (3.00)	4.16 (14.73)	3.71 (15.78)
Total cholesterol (mg/dl)	2.40 (3.31)	2.97 (3.09)	3.07 (3.33)	1.37 (1.04)
HDL-cholesterol (mg/dl)	1.12 (3.94)	1.87 (3.35)	1.26 (3.22)	3.15 (1.82)
LDL-cholesterol (mg/dl)	2.30 (3.06)	2.42 (1.8)	2.60 (1.34)	1.43 (1.28)
Triglycerides (mg/dl)	1.02 (3.31)	1.04 (0.98)	1.05 (1.03)	0.96 (0.58)
ALT (IU/L)	23.00 (41.00)	28.00 (41.10)	33.00 (42.93)	25.44 (5.62)
AST (IU/L)	43.00 (74.00)	55.00 (78.00)	55.00 (25.00)	26.00 (41.00)
TBil (ummol/L)	11.00 (23.00)	14.00 (24.00)	15.00 (4.90)	29.00 (60.00)
D-BIL (ummol/L)	1.50 (3.70)	2.27 (5.10)	2.60 (4.90)	13.00 (21.58)
ApoA (g/L)	1.21 (3.52)	1.25(3.23)	1.37 (4.09)	7.43 (88.34)
ApoB (g/L)	1.08 (0.38)	1.05(0.35)	1.04(0.38)	1.40(4.49)
*Echocardiography data before PPCI*				
LAD (mm)	35.00 (5.00)	35 (5.00)	35.00 (5.00)	35.00 (5.00)
IVSd (mm)	10.00 (1.00)	10.00 (8.00)	10 (1.00)	7.00 (8.00)
LVEDV (mm)	49 (6.00)	49.00 (6.00)	49.00 (6.00)	49.00 (6.00)
LVPWs (mm)	9.00 (1.00)	9.00 (1.00)	9.00 (1.00)	9.00 (1.00)
EF (%)	55.00 (10.00)	56 (0.00)	55.00 (42.92)	56.00 (10.00)
*Discharge medication regimen*				
Statin (%(*n*))	81.5 (2921)	76.0 (1465)	72.2 (1307)	65.6 (747)
Aspirin (%(*n*))	64.7 (2321)	76.8 (1479)	61.2 (1107)	67.0 (762)
Clopidogrel (%(*n*))	66 (2367)	52.3 (1007)	54.9 (993)	42.9 (488)
Ticagrelor (%(*n*))	20.1 (721)	27.9 (537)	21.9 (397)	27.0 (307)
Warfarin (%(*n*))	0.4 (15)	0.3 (6)	0.4 (8)	0.2 (2)
ACEI (%(*n*))	56.1 (2011)	54.0 (1040)	51.6 (933)	47.6 (542)
ARB (%(*n*))	6.7 (242)	6.3 (122)	6.7 (122)	5.9 (67)
Beta-blockers (%(*n*))	76 (2726)	71.1 (1370)	67.6 (1222)	62.0 (706)
Ezetimibe (%(*n*))	0.7 (26)	1.1 (21)	0.9 (17)	1.2 (14)
Diuretic (%(*n*))	26 (931)	21.9 (422)	22.6 (409)	18.6 (212)
*Endpoint events*				
MACE (%(*n*))	8.2 (293)	4.9 (94)	5.9 (107)	4.1 (47)
Death (%(*n*))	6 (215)	3.1 (59)	3.9 (70)	2.3 (26)
CV death (%(*n*))	4.8 (170)	1.9 (37)	2.9 (53)	1.2 (13)
Stroke (%(*n*))	1.9 (68)	1.3 (26)	1.7 (31)	1.5 (17)

Continuous data are presented as mean ± SD; categorical variables are presented as % (*n*). cTnI, cardiac troponin I; PPCI, primary percutaneous coronary intervention; BMI, body mass index; PCI, primary percutaneous coronary intervention; CABG, coronary artery bypass grafting; TC, total cholesterol; HDL-C, high-density lipoprotein cholesterol; LDL-C, low-density lipoprotein cholesterol; TG, triglyceride; ALT, alanine aminotransferase; AST, aspartate aminotransferase; TBIL, total bilirubin; D-BIL, direct bilirubin; ApoA, apolipoprotein A; ApoB, apolipoprotein B; LAD, left atrial diameter; IVSd, interventricular septal thickness diameter; LVEDV, left ventricular end systolic volume; LVPWs, left ventricular posterior wall thickness; EF, ejection fraction; ACEI, angiotensin-converting enzyme inhibitor; ARB, angiotensin receptor blocker; MACE, major adverse cardiovascular events; CV death, cardiovascular death.

**Table 2:** Characteristics of the △cTnI cohort versus cohort lack of repeated cTnI assessments.

Variables	△cTnI cohort	Cohort lack of repeated cTnI assessments	*p* value
N	1138	2448	----
Age (years)	59(12.31)	59(12.08)	0.095
Male (%(*n*))	68.5(779)	79.0(1934)	≤0.001
BMI (kg/m^2^)	25.99(3.76)	26.01(7.85)	0.917
Heart rate (beats per minute)	77.45(36.81)	77.84(25.11)	0.712
Systolic blood pressure (mmHg)	124(40)	120(72)	0.015
Diastolic blood pressure (mmHg)	77(25)	71(20)	≤0.001
History of PCI (%(*n*))	9.8(111)	12.5(306)	0.019
History of CABG (%(*n*))	0.9(10)	0.8(20)	0.845
Hypertension (%(*n*))	59.3(675)	59.7(1461)	0.855
Hyperlipidemia (%(*n*))	64.9(739)	83.5(2043)	≤0.001
Smoking (%(*n*))	38.5(438)	59.9(1465)	≤0.001
Diabetes (%(*n*))	32.2(366)	32.6(799)	0.561
Total cholesterol (mg/dl)	1.37(1.04)	2.54(2.05)	≤0.001
HDL-cholesterol (mg/dl)	3.15(1.82)	2.35(2.03)	≤0.001
LDL-cholesterol (mg/dl)	1.43(1.28)	2.35(2.03)	≤0.001
Triglycerides (mg/dl)	0.96(0.58)	1.19(0.97)	≤0.001
ALT (IU/L)	25.44(5.62)	25.99(3.79)	0.619
Statin (%(*n*))	65.6(747)	88.8(2174)	≤0.001
Aspirin (%(*n*))	67.0(762)	63.6(1559)	0.060
Clopidogrel (%(*n*))	42.9(488)	76.8(1879)	≤0.001
Ticagrelor (%(*n*))	27.0(307)	16.9(414)	≤0.001
Warfarin (%(*n*))	0.2(2)	0.5(13)	0.167
ACEI (%(*n*))	47.6(542)	60.0(1469)	≤0.001
ARB (%(*n*))	5.9(67)	7.1(175)	0.174
Beta-blockers (%(*n*))	62.0(706)	82.5(2020)	≤0.001
Ezetimibe (%(*n*))	1.2(14)	0.5(12)	0.020
Diuretic (%(*n*))	18.6(212)	29.3(719)	≤0.001
MACE (%(*n*))	4.1(47)	10.0(246)	≤0.001
Death (%(*n*))	2.3(26)	7.7(189)	≤0.001
CV death (%(*n*))	1.2(13)	6.4(157)	≤0.001
Stroke (%(*n*))	1.5(17)	2.1(51)	0.292

Continuous data are presented as mean ± SD or median (interquartile range); categorical variables are presented as % (*n*). PCI, percutaneous coronary intervention; BMI, body mass index; CABG, coronary artery bypass grafting; TC, total cholesterol; HDL-C, high-density lipoprotein cholesterol; LDL-C, low-density lipoprotein cholesterol; TG, triglyceride; ALT, alanine aminotransferase; ACEI, angiotensin-converting enzyme inhibitor; ARB, angiotensin receptor blocker; MACE, major adverse cardiovascular events; CV death, cardiovascular death.

**Table 3:** Correlation analysis: associations between patient characteristics and cTnI on admission prior PPCI, peak troponin levels after PPCI, and the △cTnI level difference before and after PPCI.

	cTnI on admission prior PPCI		Peak troponin levels after PPCI		△cTnI level difference before and after PPCI	
	Correlation coefficient	*p* value	Correlation coefficient	*p* value	Correlation coefficient	*p* value
Time from symptom onset to admission	0.432	<0.001^*∗*^	−0.004	0.880	−0.161	<0.001^*∗*^
TIMI flow grade prior PPCI	0.046	0.059	−0.206	≤0.001^*∗*^	−0.214	<0.001^*∗*^
Minimum vessel diameter prior PPCI	0.014	0.552	−0.065	0.010^*∗*^	−0.214	<0.001^*∗*^
The use of IABP	0.036	0.136	0.025	0.313	0.056	0.081
GRACE score	0.070	0.011^*∗*^	0.046	0.148	0.017	0.637
Number of stents	−0.037	0.122	<0.001	0.985	−0.028	0.383
Target lesion types	−0.032	0.166	0.021	0.368	0.091	0.005^*∗*^
The status of target organ thrombosis	−0.053	0.034^*∗*^	0.160	<0.001^*∗*^	0.154	<0.001^*∗*^
The target organ completed occlusion or not	−0.068	0.005^*∗*^	0.197	<0.001^*∗*^	0.203	<0.001^*∗*^
The target lesion involves branches or not	0.063	0.009^*∗*^	0.006	0.816	−0.031	0.335
The target organ calcified or not	−0.034	0.165	−0.031	0.224	0.025	0.446
Minimum vessel diameter after PPCI	−0.021	0.386	0.018	0.477	0.019	0.563
TIMI flow grade after PPCI	0.026	0.280	0.050	0.048^*∗*^	−0.001	0.966
LAD on discharge	0.051	0.046^*∗*^	0.135	<0.001^*∗*^	0.145	<0.001^*∗*^
LVEDV on discharge	0.067	0.007^*∗*^	0.116	<0.001^*∗*^	0.139	<0.001^*∗*^
EF on discharge	−0.145	<0.001^*∗*^	−0.156	<0.001^*∗*^	−0.091	<0.001^*∗*^

cTnI, cardiac troponin I; PPCI, primary percutaneous coronary intervention; TIMI, thrombolysis in myocardial infarction; IABP, intra-aortic balloon pump; LAD, left atrial diameter; LVEDV, left ventricular end systolic volume; EF, ejection fraction.

**Table 4:** Analysis stratified according to the quartiles of delta troponin I.

Variables	Quartile I (*n* = 284) −79.724–0.347	Quartile II (*n* = 284) 0.350–4.708	Quartile III (*n* = 285) 4.845–19.073	Quartile IV (*n* = 285) 19.161–101.660	*p* value
Median of the △cTnI level (ng/ml)	−5.14	2.09	10.38	52.00	—
Age (years)	60(17)	59(15)	57(15)	59(16)	0.192
Male (%(*n*))	77.1(219)	79.6(226)	82.5(235)	83.9(239)	0.073
BMI (kg/m^2^)	25.25(4.65)	25.95(4.59)	25.90(4.47)	25.95(4.38)	0.284
Heart rate (beats per minute)	75.00(18.00)	74.00(15.00)	73.00(18.00)	76.00(19.00)	0.840
Systolic blood pressure (mmHg)	126.00(22.00)	124.00(17.00)	123.00(23.00)	122.00(25.00)	0.670
Diastolic blood pressure (mmHg)	77.00(17.00)	76.00(15.25)	77.00(17.00)	77.00(17.00)	0.534
Anterior wall myocardial infarction	140	118	99	143	<0.001^*∗*^
History of PCI (%(*n*))	9.9(28)	12.7(36)	9.8(28)	6.7(19)	0.120
History of CABG (%(*n*))	0.7(2)	0.4(1)	0.7(2)	1.8(5)	0.307
GRACE score	102(40)	103(36)	95(34)	104(36)	0.038^*∗*^
The time from chest pain to vessel reperfusion (day)	0.47(0.60)	0.35(0.50)	0.32(0.34)	0.31(0.33)	<0.001^*∗*^
*Risk factors*					
Hypertension (%(*n*))	59.5(169)	59.5(169)	55.1(157)	63.2(180)	0.277
Hyperlipidemia (%(*n*))	77.8(221)	75.4(214)	63.2(180)	43.5(124)	0.159
Smoking (%(*n*))	56.3(160)	44(125)	36.1(103)	17.2(49)	0.848
Diabetes (%(*n*))	32.7(93)	35.3(100)	30.9(88)	29.8(85)	0.536
*Laboratory examinations*					
MYO	20.00(72.16)	20.99(66.83)	47.16(229.70)	201.68(831.70)	<0.001^*∗*^
CK-MB (ng/mL)	51.50(540.15)	25.00(161.30)	56.00(189.00)	42.04(246.54)	0.023
NT-proBNP (pg/ml)	56.39(684.29)	183.60(1170)	148.90(755.28)	111.7(965.25)	0.514
CRP (mg/L)	6.70(8.86)	4.83(8.45)	5.29(9.00)	6.51(8.06)	0.085
Uric acid (mmol/L)	8.39(69.46)	5.55(70.52)	5.75(68.26)	4.68(7.67)	<0.001^*∗*^
Creatinine (ummol/L)	1.42(4.36)	2.05(4.43)	1.45(1.32)	2.07(0.91)	<0.001^*∗*^
*Echocardiography data pre-PPCI*					
LAD (mm)	35.00(4.00)	35.00(5.00)	35.50(5.00)	36.00(5.00)	0.310
IVSd (mm)	9.00(2.00)	9.00(2.00)	9.00(2.00)	9.00(2.00)	0.476
LVEDV (mm)	48.00(5.00)	49.00(6.00)	49.00(6.00)	50.00(6.00)	0.007^*∗*^
LVPWs (mm)	10.00(1.00)	9.00(1.00)	9.00(1.00)	9.00(1.00)	0.012
EF (%)	56.00(10.00)	56.00(9.00)	57.00(7.00)	54.00(10.00)	<0.001^*∗*^
*Angiographic findings pre-PPCI*					
Triple-vessel disease (%(*n*))	34.2(97)	32.4(92)	33.3(95)	33.0(94)	0.984
Length of target lesions (mm)	24.00(17.00)	24.00(17.00)	25.00(16.25)	23.5(16.00)	0.479
Target lesion types = *C* (%(*n*))	59.5(169)	62.7(178)	65.3(186)	69.5(198)	0.096
The status of target organ thrombosis (%(*n*))	38.7(110)	44.3(126)	57.9(165)	57.9(165)	0.002^*∗*^
The target organ completed occlusion (%(*n*))	48.6(138)	54.9(156)	69.8(199)	73.3(209)	<0.001^*∗*^
The target lesion involves branches (%(*n*))	37.3(106)	30.6(87)	31.6(90)	27.1(77)	0.150
The target organ calcification (%(*n*))	44.0(125)	44.0(125)	42.2(120)	43.6(124)	0.957
TIMI flow grade prior PPCI = 0 (%(*n*))	51.4(146)	60.2(171)	73.0(208)	77.2(220)	<0.001^*∗*^
Minimum vessel diameter prior PPCI (mm)	0.00(0.175)	0.00(0.15)	0.00(0.035)	0.00(0.00)	<0.001^*∗*^
*Procedural data*					
The use of IABP (%(*n*))	6.0(17)	3.2(9)	4.2(12)	6.7(19)	0.100
Number of cardiac stents>1 (%(*n*))	26.8(76)	21.9(62)	26.6(67)	23.6(67)	0.272
No-reflow phenomenon (%(*n*))	2.8(8)	3.6(10)	3.2(9)	3.2(9)	0.377
Minimum vessel diameter after PPCI (mm)	3.00(1.00)	3.00(1.00)	3.00(1.00)	3.00(1.00)	0.949
*Discharge medication regimen*					
Statin (%(*n*))	86.3(245)	75.0(213)	66.0(188)	35.4(101)	0.009^*∗*^
Aspirin (%(*n*))	88.7(252)	77.8(221)	66.3(189)	35.1(100)	<0.001^*∗*^
Clopidogrel (%(*n*))	59.9(170)	52.1(148)	38.2(109)	21.4(61)	0.028^*∗*^
Ticagrelor (%(*n*))	33.1(94)	29.9(85)	29.5(84)	15.4(44)	0.445
ACEI (%(*n*))	63.7(181)	57.7(164)	46.3(132)	22.8(65)	0.023^*∗*^
ARB (%(*n*))	7.4(21)	5.6(16)	5.6(16)	4.9(14)	0.468
Beta-blockers (%(*n*))	83.1(236)	73.2(208)	58.9(168)	33.0(94)	0.024^*∗*^
Ezetimibe (%(*n*))	2.5(7)	0.7(2)	1.4(4)	0.4(1)	0.370
Nitrate (%(*n*))	75.4(214)	63.7(181)	52.3(149)	26.7(76)	0.002^*∗*^
Diuretic (%(*n*))	26.8(76)	20.1(57)	15.4(44)	12.3(35)	0.300
*Clinical outcomes evaluated during the follow-up*					
MACE (%(*n*))	2.1(6)	6.7(19)	3.9(11)	3.9(11)	0.052
Death (%(*n*))	1.4(4)	2.8(8)	1.8(5)	3.2(9)	0.342
CV death (%(*n*))	0.8(2)	2.2(6)	0.7(2)	1.1(3)	0.579
Recurrent myocardial infarction	1.1(3)	2.8(8)	2.1(6)	2.1(6)	0.225
Angina pectoris	5.7(16)	7.1(20)	7.4(21)	3.9(11)	0.575
Stroke (%(*n*))	1.4(4)	2.1(6)	1.4(4)	1.1(3)	0.893

Continuous data are presented as mean ± SD or median (interquartile range); categorical variables are presented as % (*n*). cTnI, cardiac troponin I; BMI, body mass index; PCI, primary percutaneous coronary intervention; CABG, coronary artery bypass grafting; TC, total cholesterol; HDL-C, high-density lipoprotein cholesterol; LDL-C, low-density lipoprotein cholesterol; TG, triglyceride; ALT, alanine aminotransferase; AST, aspartate aminotransferase; TBIL, total bilirubin; D-BIL, direct bilirubin; ApoA, apolipoprotein A; ApoB, apolipoprotein B; LAD, left atrial diameter; IVSd, interventricular septal thickness diameter; LVEDV, left ventricular end systolic volume; LVPWs, left ventricular posterior wall thickness; EF, ejection fraction; ACEI, angiotensin-converting enzyme inhibitor; ARB, angiotensin receptor blocker.

**Table 5:** Results of the univariate and multivariable Cox proportional hazards model applied to assess correlates of 2-year MACE.

Baseline parameters	Univariate Cox regression		Multivariate Cox regression	
	HR (95% CI)	*p* value	HR (95% CI)	*p* value
△cTnI	1.013 (1.004–1.022)	0.005^*∗*^	1.018 (1.001–1.035)	0.042^*∗*^
Q1△cTnI	Ref	Ref	Ref	Ref
Q2△cTnI	3.399 (1.357–8.514)	0.009^*∗*^	4.080 (1.342–12.403)	0.013^*∗*^
Q3△cTnI	2.637 (0.971–7.162)	0.057	2.561 (0.744–8.819)	0.136
Q4△cTnI	3.583 (1.317–9.752)	0.012^*∗*^	1.566 (0.290–8.444)	0.602
BMI	0.951 (0.876–1.033)	0.234	0.945 (0.863–1.035)	0.223
Anterior wall myocardial infarction	1.255 (0.709–2.224)	0.436	1.315 (0.681–2.540)	0.414
Uric acid	0.984 (0.974–0.994)	0.002^*∗*^	0.987 (0.977–0.998)	0.018^*∗*^
Number of stents	0.776 (0.477–1.261)	0.306	1.152 (0.697–1.905)	0.581
No-reflow phenomenon	1.014 (0.139–7.387)	0.989	1.463 (0.190–11.259)	0.715
Target lesion types				
A	Ref	Ref	Ref	Ref
B1	0.272 (0.091–0.813)	0.020^*∗*^	—	0.908
B2	0.103 (0.031–0.340)	<0.001^*∗*^	—	0.917
C	0.162 (0.067–0.393)	<0.001^*∗*^	—	0.915
Organ thrombosis	1.011(0.622–1.643)	0.965	0.998 (0.481–2.075)	0.988
Completed occlusion	0.990 (0.524–1.872)	0.977	0.864 (0.411–1.820)	0.701
Target lesion involving branches	0.930 (0.481–1.797)	0.829	0.943 (0.456–1.952)	0.875

MACE, major adverse cardiovascular events; HR, hazard ratio; cTnI, cardiac troponin I; PPCI, primary percutaneous coronary intervention; BMI, body mass index.

## Data Availability

The datasets uesd and/or analyzed during this study are available from the corresponding author on reasonable request.
